# Supplementary material for: AffyMAPSDetector: a software tool to characterize Affymetrix GeneChip™ expression arrays with respect to SNPs
Source: BMC Bioinformatics. 2007 Jul 30;8:276. doi: 10.1186/1471-2105-8-276 (PMC1959249; doi:10.1186/1471-2105-8-276)
Supplement: Additional file 13 — AffyMAPSDetector v1 source code. AffyMAPSDetector source code. [file 1471-2105-8-276-S13.zip › AffyMAPSDetector_v1_src/doc/jwf/class-use/WizardListener.html]

Uses of Interface jwf.WizardListener


|  |  |  |  |
| --- | --- | --- | --- |
| **Overview** \| **Package** \| **Class** \| **Use** \| **Tree** \| **Deprecated** \| **Index** \| **Help** | | |  |
| PREV   NEXT | **FRAMES**    **NO FRAMES**     **All Classes** |  |  |


---


## **Uses of Interface jwf.WizardListener**

|  |  |
| --- | --- |
| Packages that use WizardListener | |
| **affychip.snpdetector.ui** |  |
| **affychip.snpdetector.util** |  |
| **jwf** | This package contains classes that can be used to create and run a Wizard. |

Uses of WizardListener in affychip.snpdetector.ui

|  |  |
| --- | --- |
| Classes in affychip.snpdetector.ui that implement WizardListener | |
| `class` | `FileLoaderWizardDialog`             Title: Probe SNP Detector |

Uses of WizardListener in affychip.snpdetector.util

|  |  |
| --- | --- |
| Classes in affychip.snpdetector.util that implement WizardListener | |
| `class` | `GeneAnnotLoaderWizard` |

Uses of WizardListener in jwf

|  |  |
| --- | --- |
| Classes in jwf that implement WizardListener | |
| `class` | `WizardAdapter`             This class provides means of abreviating work when using the WizardListener allowing the developer to implement only the needed methods |

|  |  |
| --- | --- |
| Methods in jwf with parameters of type WizardListener | |
| `void` | `Wizard.addWizardListener(WizardListener listener)`             Add a listener to this wizard. |
| `void` | `Wizard.removeWizardListener(WizardListener listener)`             Remove a listener from this wizard. |

---


|  |  |  |  |
| --- | --- | --- | --- |
| **Overview** \| **Package** \| **Class** \| **Use** \| **Tree** \| **Deprecated** \| **Index** \| **Help** | | |  |
| PREV   NEXT | **FRAMES**    **NO FRAMES**     **All Classes** |  |  |


---
